# Time Reversal Acoustic Communication Using Filtered Multitone Modulation

**DOI:** 10.3390/s150923554

**Published:** 2015-09-17

**Authors:** Lin Sun, Baowei Chen, Haisen Li, Tian Zhou, Ruo Li

**Affiliations:** 1 College of Underwater Acoustic Engineering, Harbin Engineering University, Harbin 150001, China; E-Mails: zcbsunny@hrbeu.edu.cn (L.S.); chenbaowei@hrbeu.edu.cn (B.C.); zhoutian@hrbeu.edu.cn (T.Z.); liruo@hrbeu.edu.cn (R.L.); 2 Science and Technology on Underwater Acoustic Laboratory, Harbin Engineering University, Harbin 150001, China

**Keywords:** underwater acoustic communication, filtered multitone modulation, time reversal, adaptive equalization

## Abstract

The multipath spread in underwater acoustic channels is severe and, therefore, when the symbol rate of the time reversal (TR) acoustic communication using single-carrier (SC) modulation is high, the large intersymbol interference (ISI) span caused by multipath reduces the performance of the TR process and needs to be removed using the long adaptive equalizer as the post-processor. In this paper, a TR acoustic communication method using filtered multitone (FMT) modulation is proposed in order to reduce the residual ISI in the processed signal using TR. In the proposed method, FMT modulation is exploited to modulate information symbols onto separate subcarriers with high spectral containment and TR technique, as well as adaptive equalization is adopted at the receiver to suppress ISI and noise. The performance of the proposed method is assessed through simulation and real data from a trial in an experimental pool. The proposed method was compared with the TR acoustic communication using SC modulation with the same spectral efficiency. Results demonstrate that the proposed method can improve the performance of the TR process and reduce the computational complexity of adaptive equalization for post-process.

## 1. Introduction

For the construction of underwater acoustic sensor networks, high data rate acoustic communications have been extensively researched to improve the throughput of sensor nodes. In order to achieve reliable performance with relatively low implementation complexity, the high data rate single-carrier (SC) acoustic communication using time reversal (TR) has been studied in recent years [1,2,3,4,5,6,7]. For a given underwater acoustic channel with large multipath spread, the higher symbol rate results in the larger intersymbol interference (ISI) span which reduces the performance of the TR process. The larger ISI span needs to be removed through longer adaptive equalizers in the high data rate SC acoustic communication using TR.

To shorten the span of ISI in SC acoustic communication using TR, multicarrier modulation can be used in the TR acoustic communication. In multicarrier modulation for acoustic communications [8,9,10,11,12,13,14,15,16,17,18,19,20,21,22,23], researches have mainly focused on orthogonal frequency-division multiplexing (OFDM) [8,9,10,11,12,13,14,15,16,21,22,23]. In OFDM, the available frequency band is divided into a number of overlapping subcarriers which have the small bandwidth to avoid ISI. However, since the subcarriers in OFDM are overlapping, the small bandwidth of each subcarrier can result in that the communication performance is sensitive to frequency offset. A slight frequency offset in OFDM can lead to severe intercarrier interference (ICI). Therefore, in order to obtain good performance, complicated algorithms at the receiver must be exploited to suppress ICI [24,25,26]. In order to avoid ICI, some other multicarrier modulation schemes have been studied in acoustic communications. Zhang *et al.* [17] proposed a multicarrier modulation scheme which divided the available frequency band into two separate subcarriers with 1 kHz bandwidth, and a guard band was set between subcarriers. A similar multicarrier modulation scheme with wider subcarriers was proposed by Song *et al.* [18,19], in which the bandwidth of each subcarrier was 4.5 kHz and guard bands were also inserted between subcarriers. In the multicarrier modulation schemes respectively proposed by Zhang *et al.* and Song *et al.*, widely separate subcarriers and guard bands can keep the acoustic communication system from ICI, but guard bands can also result in the lower spectral efficiency compared with the SC modulation using similar transmit filters with the same roll-off factor over the whole frequency band.

In order to overcome the disadvantage of OFDM while keeping high spectral efficiency, Gomes *et al.* [20] studied the acoustic communication using filtered multitone (FMT) modulation [27,28,29,30,31,32,33] which divided the available frequency band into several separate subcarriers without guard bands, and compared the performance of FMT acoustic communication with that of OFDM acoustic communication. In FMT, each subcarrier is separate and the bandwidth is relatively wide. Therefore, when some frequency offset occurs in FMT, ICI is relatively slight and a minor concern compared with ISI. Furthermore, compared with the multicarrier modulation schemes proposed respectively by Zhang *et al.* and Song *et al.*, due to no guard bands, FMT has the same spectral efficiency as the SC modulation using similar transmit filters with the same roll-off factor over the whole frequency band. FMT modulation for acoustic communications has attracted increasing interest recently [31,32].

Based on the high spectral efficiency and good ICI suppression of FMT modulation, a TR acoustic communication method using FMT modulation is proposed in this paper for the purpose of improving the performance of the TR process and reducing the computational complexity of adaptive equalization for post-process. In the proposed method, FMT modulation is exploited to modulate information symbols onto separate subcarriers for parallel transmission, and TR technique, as well as adaptive equalization, is adopted at the receiver to suppress ISI and noise. The performance of the proposed method is assessed through simulation and real data from a trial in an experimental pool with the frequency band of 7.5–15.5 kHz. Performance indicators of the proposed method are compared with that of the TR acoustic communication using SC modulation with the same spectral efficiency, and results show the validity of the proposed method.

The contribution of this paper consists of three dimensions: (1) aiming at the issue that the large ISI span in the high data rate TR acoustic communication using SC modulation decreases the performance of the TR process and increases the computational complexity of equalization for post-processing, the TR acoustic communication method using FMT modulation is proposed; (2) the system model of the proposed method including the transmit structure and the receive structure are presented, and verified using theory analysis; and (3) through simulation and experiment, the performance of the proposed method is assessed and compared with that of the TR acoustic communication using SC modulation, and results confirm that the proposed method improves the performance of the TR process and reduces the computational complexity of adaptive equalization for post-process.

The paper is organized as follows. Section 2 presents the system model of the proposed method including the transmit structure and the receive structure, and analyzes the system model in theory. Section 3 assesses the performance of the proposed method through simulation and real data from a trial in an experimental pool, and compares the performance of the proposed method with that of the TR acoustic communication using SC modulation. Finally, concluding remarks are given in Section 4.

## 2. System Model of the TR Acoustic Communication Using FMT

### 2.1. Transmit Structure

**Figure 1:**
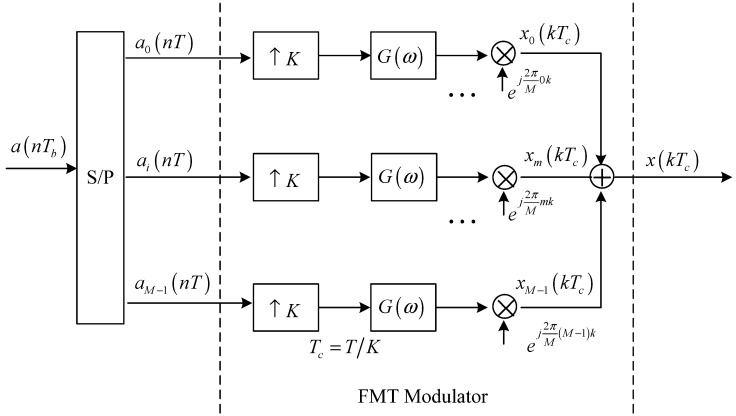
Block diagram for the transmit structure of the proposed method.

The block diagram for the transmit structure based on baseband model is displayed in Figure 1. The information symbols $a\left(nT_{b}\right)$ with the symbol interval $T_{b}$ are firstly processed by a serial-to-parallel converter. Each output signal $a_{i}\left(nT\right),i=1,\ldots ,M$, of the serial-to-parallel converter is then processed by a *K*-times up-sampler, transmit filter and carrier modulator. Through combining all modulated signals, the transmit FMT signal can be obtained finally.

**Figure 2:**
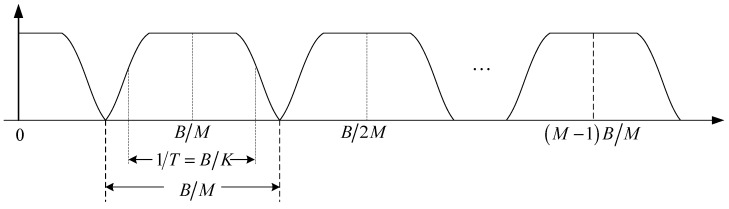
The complex baseband spectrum of the transmit FMT signal.

Referring to Figure 1, the transmit FMT signal can be expressed in complex baseband as:

(1) x(kTc)=∑i=1M∑n=−∞+∞a(nKTc)ig(kTc−nKTc)ej2πMik

where $T_{c}=T/K$ denotes the symbol interval after up-sampling and $g\left(kT_{c}\right)$ denotes the time domain response of the transmit filter G(ω). To obtain the highest spectrum efficiency, the transmit filter should be an ideal low-pass filter. However, since the ideal low-pass filter cannot be achieved, in fact, the transmit filter must be designed to strike a balance between the implementation complexity and the spectral containment [28,33]. In the proposed method, we follow the transmit filter design scheme proposed by Wilbur *et al.* [28] which selects the root raised cosine (RRC) shaping filter for transmit filtering. Figure 2 shows the complex baseband spectrum of the transmit FMT signal $x\left(kT_{c}\right)$ when the RRC shaping filter is used. Referring to Figure 2, the entire band *B* is divide into *M* subcarriers, the occupying bandwidth of each subcarrier is B/M, the effective bandwidth of each subcarrier is B/K, and the roll-off factor of the RRC shaping filter is α=K/M−1. Since α must be in the range of [0, 1], K can change from M to 2M for a given M to compromise between ICI/ISI sensitivity and the symbol rate. Figure 2 indicates that since the maximum side-lobe peak of the transmit filter in the FMT modulator is designed very close to zero in practice, the effect of ICI can be neglected in the stable acoustic channel. Furthermore, since the subcarriers in FMT have relatively wide bandwidth, ICI also is a minor concern compared with ISI in the acoustic channel with realistic Doppler shift.

### 2.2. Receive Structure

#### 2.2.1. FMT Demodulation

For the convenient expression, the symbol interval $T_{c}$, which has been explained in Section 2.1, is omitted in the following sections. After x(k) is transmitted through the acoustic channel H(ω), the received FMT signal r(k) can be expressed as:

(2) $$r\left(k\right)=\sum_{p=-\infty }^{+\infty }x\left(k-p\right)h\left(p\right)+w\left(k\right)$$

where h(k) denotes the time domain response of the acoustic channel H(ω), and w(k) denotes the channel noise.

**Figure 3:**
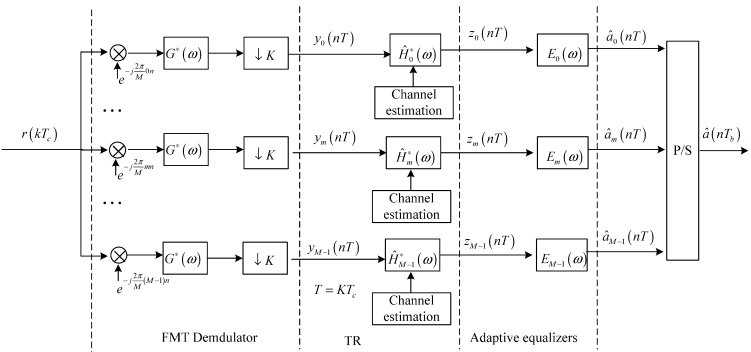
Block diagram for the receive structure of the proposed method.

Figure 3 shows the block diagram for the receive structure based on baseband model. Referring to Figure 3, it is observed that the FMT demodulator is composed of subcarrier demodulators, receive filters and *K*-times down-samplers. The *m*-th output signal of the FMT demodulator can be expressed as:

(3) ym(nK)=∑i=1M∑l=−∞+∞∑k=−∞+∞∑p=−∞+∞g(k−p−lK)h(p)e−j2πMipg′(nK−k)ej2πM(i−m)ka(lK)i+∑k=−∞+∞w(k)g′(nK−k)e−j2πMmk=∑i=1M∑l=−∞+∞f(m,i)(l,n)a(lK)i+ηm(nK)

where $g^{\prime }\left(k\right)$ denotes the time domain response of the receive filter $G^{*}\left(\omega \right)$, $f^{\left(m,i\right)}\left(l,n\right)$ denotes the time domain response of the composite channel between the *i*-th input signal of FMT modulator a(nK)i and the *m*-th output signal of FMT demodulator $y_{m}\left(nK\right)$, and $\eta_{m}\left(nK\right)$ denotes the output noise in the *m*-th output signal of FMT demodulator. The corresponding frequency domain expressions of $f^{\left(m,i\right)}\left(l,n\right)$ and $\eta_{m}\left(nK\right)$ are:

(4) $$F^{\left(m,i\right)}\left(\omega \right)=G\left(\omega \right)H\left(\omega +\frac{2\pi }{M}i\right)G^{*}\left(\omega \text{+}\frac{2\pi i}{M}-\frac{2\pi m}{M}\right)$$

(5) $$\eta_{m}\left(\omega \right)=W\left(\omega \right)G^{*}\left(\omega -\frac{2\pi }{M}m\right)$$

#### 2.2.2. TR Process

It is observed from Equations (3) and (4) that the channel response for the *i*-th subcarrier is:

(6) $$H_{i}\left(\omega \right)=H\left(\omega +\frac{2\pi }{M}i\right)$$

Equation (6) shows that the channel responses for all the subcarriers are different from each other. Therefore, in order to process the output signals of FMT demodulator using TR technique, the channel response of each subcarrier should be estimated individually.

From Figure 3, it is observed that the output signal of the *m*-th matched filter ${\hat{H}}_{m}^{*}\left(\omega \right)$ can be expressed as:

(7) zm(nK)=∑i=1M∑l=−∞+∞∑λ=−∞+∞∑k=−∞+∞∑p=−∞+∞g(k−p−lK)hi(p)g′(λK−k)h′^m(nK−λK)ej2πM(i−m)ka(lK)i+∑λ=−∞+∞ηm(λK)h′^m(nK−λK)=∑i=1M∑l=−∞+∞q(m,i)(l,n)a(lK)i+ζm(nK)

where $q^{\left(m,i\right)}\left(l,n\right)$ denotes the time domain response of the composite channel between a(nK)i, the *i*-th input signal of FMT modulator and $z_{m}\left(nK\right)$, the *m*-th output signal of the TR process, ${\hat{h^{\prime }}}_{m}\left(nK\right)$ denotes the time domain response of the matched filter ${\hat{H}}_{m}^{*}\left(\omega \right)$, and $\zeta_{m}\left(nK\right)$ denotes the output noise of the TR process. The frequency domain expressions of $q^{\left(m,i\right)}\left(l,n\right)$ and $\zeta_{m}\left(nK\right)$ are:

(8) $$Q^{\left(m,i\right)}\left(\omega \right)=G\left(\omega \right)H_{i}\left(\omega \right)G^{*}\left(\omega \text{+}\frac{2\pi i}{M}-\frac{2\pi m}{M}\right){\hat{H}}_{m}^{*}\left(\omega \text{+}\frac{2\pi i}{M}-\frac{2\pi m}{M}\right)$$

(9) $$\zeta_{m}\left(\omega \right)=W\left(\omega \right)G^{*}\left(\omega -\frac{2\pi }{M}m\right){\hat{H}}_{m}^{*}\left(\omega -\frac{2\pi }{M}m\right)$$

For the convenient analysis, the $z_{m}\left(nK\right)$ in Equation (7) can be further expressed as:

(10) zm(nK)=q(m,m)(n,n)a(nK)m+∑l=−∞l≠n+∞q(m,m)(l,n)a(lK)m+∑i=1i≠mM∑l=−∞+∞q(m,i)(l,n)a(lK)i+ζm(nK)

Equation (10) indicates that the interference in output signals of the TR process is composed by ISI, ICI, and noise. Combining Equation (10) with Equation (8), it is concluded that since ICI can be considered as zero in the stable acoustic channel, ISI is the main factor degrading the performance of the TR process when the transmitted symbol energy is high enough.

It is indicated from Equations (4) and (8) that since the response of each filter for the TR process is matched to the estimated response of channel for each subcarrier, ISI can be compressed using the TR process. However, it should be mentioned that since the frequency domain response of the *m*-th filter for the TR process is ${\hat{H}}_{m}^{*}\left(\omega \right)$ but not ${\hat{H}}_{m}^{-1}\left(\omega \right)$, residual ISI is still existing even when the channel is estimated without any error. Residual ISI reduces the performance of the TR process.

#### 2.2.3. Adaptive Equalization

In order to mitigate residual ISI further, adaptive equalization shown in Figure 3 is used in the proposed method for post-process. The adaptive equalizer used for each subcarrier is the linear equalizer to avoid error propagation, and the output signal of the *m*-th adaptive equalizer $E_{m}\left(\omega \right)$ can be expressed as:

(11) $${\hat{a}}_{m}\left(nK\right)=\sum_{i=-N_{1}}^{N_{2}}d_{m}^{i}z_{m}\left(nK-iK\right)$$

where $d_{m}^{i}$ denotes the *i*-th tap coefficient of the *m*-th adaptive equalizer $E_{m}\left(\omega \right)$ with $N_{1}+N_{2}+1$ taps. The number of taps, $N_{1}+N_{2}+1$, is related to the span of ISI in the composite channel. The larger the span of ISI is, the bigger the number of taps used in the adaptive equalizer is. Since the FMT modulation exploited in the proposed method can widen the symbol interval of each subcarrier and reduce the span of ISI in each composite channel $Q^{\left(m,i\right)}\left(\omega \right)$, the taps of adaptive equalizer for each subcarrier in the proposed method decrease.

In the proposed method, the recursive least square (RLS) algorithm is selected to adjust the tap coefficients of each adaptive equalizer, since the convergence rate of RLS is relatively fast and does not depend on the input signal. The taps of the adaptive equalizer for each subcarrier are adjusted separately. When the total number of the taps for adaptive equalization in the proposed method is equal to that of the TR acoustic communication using SC modulation, computational complexity of adaptive equalization in the proposed method is lower than that of the TR acoustic communication using SC modulation.

Referring to Figure 3, the output signals of all adaptive equalizers are processed by a parallel-to-serial converter, and then the estimation of the transmit symbols $\hat{a}\left(nT_{b}\right)$ can be obtained.

## 3. Performance Assessment

### 3.1. Simulation

#### 3.1.1. Channel Model

This subsection introduces channel model used in simulation. The channel model was constructed based on the multipath geometry [1,34]. The frequency response of the shallow water channel can be expressed as:

(12) $$H\left(\omega \right)=\sum_{p=1}^{P}c_{p}e^{-j\omega \tau_{p}}$$

where P denotes the number of multipath arrivals, $c_{p}$ denotes the gain of the *p*-th propagation path, and $\tau_{p}$ denotes the delay of the *p*-th propagation path. Both $c_{p}$ and $\tau_{p}$ are computed from the length of the *p*-th propagation path $l_{p}$. The path delay $\tau_{p}$ is computed as $\tau_{p}=l_{p}/c$, where c denotes the sound speed. The phase of path gain $c_{p}$ is calculated as $∠c_{p}=-2\pi f_{c}\tau_{p}$, where $f_{c}$ denotes carrier frequency. The magnitude of $c_{p}$ is computed as $\left|c_{p}\right|={\text{Γ}}_{p}/\sqrt{A\left(l_{p}\right)}$, where ${\text{Γ}}_{p}$ denotes the magnitude of cumulative reflection coefficient along the *p*-th propagation path and $A\left(l_{p}\right)$ denotes the nominal acoustic propagation loss of the *p*-th propagation path. The magnitude of cumulative reflection coefficient ${\text{Γ}}_{p}$ is computed as ${\text{Γ}}_{p}={\left|v_{s}\right|}^{n_{s}}{\left|v_{b}\right|}^{n_{b}}$, where $\left|v_{s}\right|$ denotes the magnitude of the surface reflection coefficient, $\left|v_{b}\right|$ denotes the magnitudes of the bottom reflection coefficient, $n_{s}$ denotes the number of the surface reflection, and $n_{b}$ denotes the number of the bottom reflection. The propagation loss $A\left(l_{p}\right)$ is computed as $A\left(l_{p}\right)=l_{p}^{k}{\left[a\left(f_{c}\right)\right]}^{l_{p}}$, where k denotes the spreading factor and $a\left(f_{c}\right)$ denotes the absorption coefficient. The coefficient $a\left(f_{c}\right)$ is computed in dB/km as $10\log a\left(f_{c}\right)=0.11f_{c}^{2}/\left(1+f_{c}^{2}\right)+44f_{c}^{2}\left(4100+f_{c}^{2}\right)+2.75\times 10^{-4}f_{c}^{2}+0.0003$ when $f_{c}$ is given in kHz.

The parameters for building the shallow water channel model were set as follows. The channel was 75 m in depth, and its bottom was supposed as flat. The transmit sensor and receive sensor were individually placed at 40 m and 30 m below the sea surface, and the range between them was 3000 m. The paths which have undergone bottom reflection twice at most were considered in channel modeling. The sound speed, c, was set to 1500 m/s. The surface reflection coefficient, $\left|v_{s}\right|$, and the bottom reflection coefficient $\left|v_{b}\right|$ were set to 1 and 0.8, respectively. The spreading factor k was set to 2. The entire communication band was 7.5–15.5 kHz. The frequency of the carrier, $f_{c}$, was 11.5 kHz when the acoustic channel response corresponding to the SC modulation was computed. When the channel response corresponding to the first subcarrier of FMT modulation was computed, the frequency values of the carrier were set to 8 kHz, 7.75 kHz and 7.625 kHz for M=8, M=16, and M=32, respectively. The channel responses for other subcarriers of FMT for M=8, M=16, and M=32 were computed based on Equation (6).

The other parameters were set as follows. Binary phase shift keying (BPSK) was used to encode information symbols. The number of information symbols is 6400, and 800 symbols ahead were used as training symbols. The linear equalizers were used for post-process to avoid error propagation. The RRC shaping filter with the roll-off factor α=0.5 was selected as the transmit filter.

#### 3.1.2. Simulation Results

Based on the shallow water channel model, simulation results are provided to assess the advantages of the proposed method over the TR acoustic communication using SC modulation.

**Figure 4:**
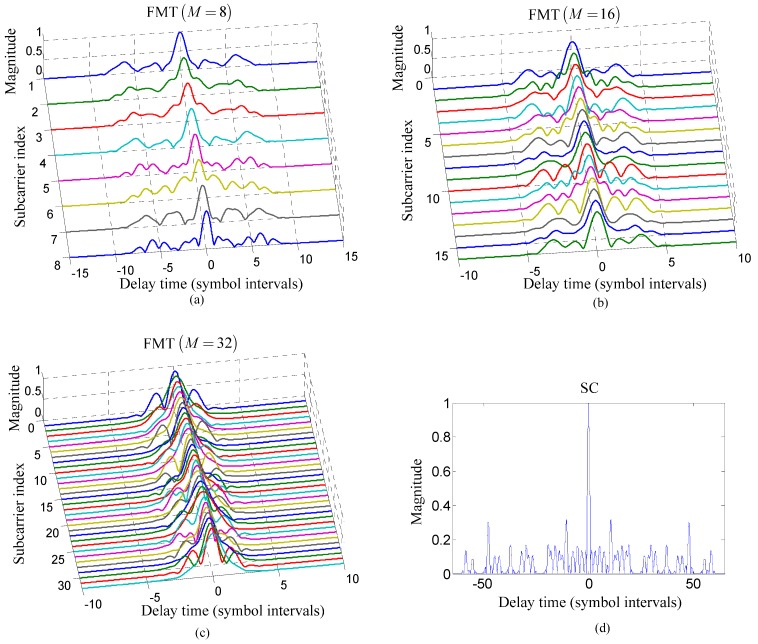
Comparison of the time domain response of each composite channel from the transmit filter to the matched filter for the TR process between two communication methods: (**a**–**c**) the proposed method for M=8, M=16, and M=32; and (**d**) the TR acoustic communication using SC modulation.

Figure 4 compares the time domain response of each composite channel from the transmit filter to the matched filter for the TR process between the proposed method and the TR acoustic communication using SC modulation. It is noticed that the span of ISI in each composite channel is reduced using the proposed method compared with the TR acoustic communication using SC modulation and, moreover, the span of ISI decreases with the increasing number of subcarriers. However, the larger number of subcarriers leads to the smaller bandwidth of each subcarrier which demands the longer transmit filter with more complicated implementation for the purpose of maintaining high spectral containment. Therefore, in practice, the number of subcarriers in FMT cannot increase indefinitely and should be selected in consideration of maintaining high ISI suppression and relatively low implementation complexity of the transmit filter.

**Figure 5:**
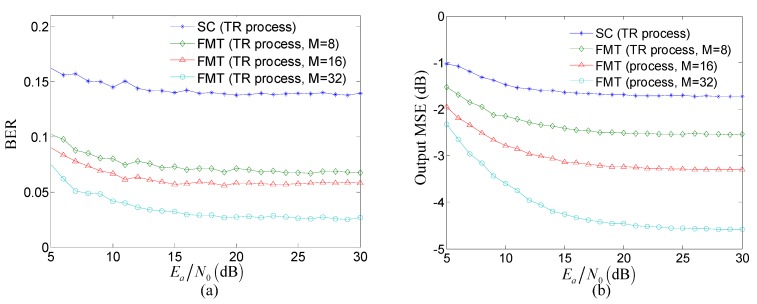
Comparison of the performance of the TR process between the proposed method and the TR acoustic communication using SC modulation: (**a**) BER; and (**b**) output MSE.

Figure 5 compares the performance of the TR process between the proposed method and the TR acoustic communication using SC modulation. Bit error rate (BER) and the output mean square error (MSE) computed by the mean square difference between the transmit symbols and the estimate of transmit symbols are used as indicators to assess the performance, and the low values of BER and MSE imply the good performance. In Figure 5, $E_{a}$ denotes the transmitted energy per symbol, and $N_{0}$ denotes the power spectral density of the channel noise w(k), and the ratio $E_{a}/N_{0}$ implies the effect of noise.

Several observations can be made from Figure 5. Firstly, the BER and output MSE of the TR process in the proposed method is obviously lower than that in the TR acoustic communication using SC modulation under the same $E_{a}/N_{0}$. The reason for this observation is that compared with the TR acoustic communication using SC modulation, the proposed method can reduce the effect of ISI through exploiting FMT to decrease the ISI span of composite channel shown in Figure 4. Therefore, when the effect of noise is the same, the performance of the TR processing in the proposed method is superior to that in the TR acoustic communication using SC modulation. Secondly, the BER and output MSE of the TR process in the proposed method decrease when the number of subcarriers increases under the same $E_{a}/N_{0}$. The reason for this observation is that in the proposed method, the ISI span of composite channel decreases with the increasing number of subcarriers. Therefore, the performance of the proposed method increased with the decreasing effect of ISI when the effect of noise is same. Thirdly, when $E_{a}/N_{0}\geq \text{20 dB}$, the BER and output MSE of the TR process in two communication methods both tends toward saturation. The reason for this observation is that when the effect of noise can be neglected due to the high value of $E_{a}/N_{0}$, the effect of ISI determined by the ISI span in composite channel shown in Figure 4 is the major factor influencing the performance. Therefore, the performance of the TR process in two communication methods both tends toward saturation as the result of high $E_{a}/N_{0}$ and fixed ISI span in composite channel for the same number of subcarriers, M.

**Figure 6:**
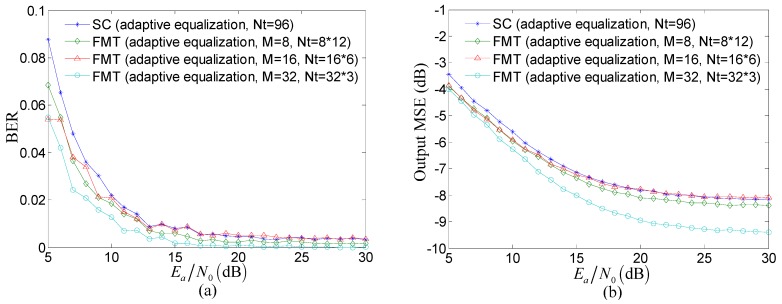
Comparison of the performance of adaptive equalization for post-process between the proposed method and the TR acoustic communication using SC modulation: (**a**) BER; and (**b**) output MSE.

Figure 6 compares the performance of adaptive equalization for post-process between the proposed method and the TR acoustic communication using SC modulation. In Figure 6, $N_{t}$ denotes the total number of taps in all adaptive equalizers. The number of adaptive equalizers equals the number of subcarriers in the proposed method while the number of adaptive equalizers equals one in the TR acoustic communication using SC modulation. The taps of each adaptive equalizer are adjusted using RLS algorithm and the number of the taps is determined by the ISI span in each composite channel corresponding to each subcarrier.

Several observations can be made from Figure 6. Firstly, the performance is further improved using adaptive equalization as post-process after the TR process. Secondly, when $E_{a}/N_{0}\geq 10$ and the total number of taps $N_{t}$ are equal, the performance of adaptive equalization in the proposed method individually for M=8, M=16, and M=32 is very close to that of the TR acoustic communication using SC modulation except that the output MSE of the proposed method for M=32 is reduced by 1 dB. Thirdly, although the total number of taps $N_{t}$ is equal, each adaptive equalizer in the proposed method is adjusted individually and therefore the computational complexity of adaptive equalization can be reduced using the proposed method compared with the TR acoustic communication using SC modulation. For RLS algorithm used in the simulation, the multiplication computation of each interaction in the TR acoustic communication using SC modulation is $O\left(96^{2}\right)$. The multiplication computation of each interaction in proposed method individually for M=8, M=16, and M=32 is $8\times O\left(12^{2}\right)$, $16\times O\left(6^{2}\right)$, and $32\times O\left(3^{2}\right)$. That is to say, the computational complexity of adaptive equalization for M=8, M=16, and M=32 is respectively cut by 7/8, 15/16, and 31/32 in the proposed method.

### 3.2. Experiment

#### 3.2.1. Experimental Setup

In order to assess the validity of the proposed method in the real underwater acoustic channel, experiments are designed and conducted in a pool. The length, width and depth of the pool were 45 m, 5 m, and 6 m, respectively. The bottom of pool was covered with sand and the sides of pool were covered with acoustic absorbent. The transmit sensor was a hemispherical transducer which was placed at 1.5 m below the surface. The receive sensor was a spherical hydrophone which was deposed at 1.5 m away from the surface. The communication distance between the transmitter and receiver was 5.5 m.

**Table 1:** Parameters for two communication methods in the experiment.

Parameters	The Proposed Method	The TR Acoustic Communication Using SC Modulation
Communication frequency band B	7.5–15.5 kHz	7.5–15.5 kHz
The number of subcarriers M	8	1
Roll-off factor α	0.5	0.5
Symbol interval T	1.5 ms	0.1875 ms
Number of total symbols	8 × 800	6400
Number of training symbol	8 × 100	800
Number of equalizers	8	1
Total number of taps $N_{t}$	8 × 8	64
Forgetting factor of RLS algorithm λ	0.999	0.999
Proportional tracking constant in DPLL $K_{1}$	0.1	0.1
Integral tracking constant in DPLL $K_{2}$	0.002	0.002
Constellation	BPSK	BPSK

**Figure 7:**
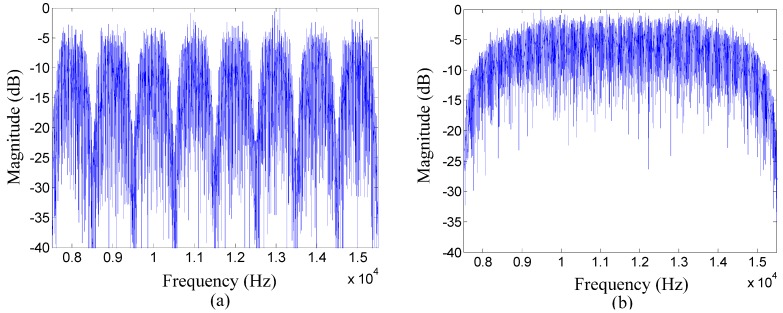
Comparison of the frequency spectrum of the information packet between two communication methods in the experiment: (**a**) the proposed method; and (**b**) the TR acoustic communication using SC modulation.

Experiment results of the proposed method are compared with that of the TR acoustic communication using SC modulation. In the experiment, parameters for the proposed method and the TR acoustic communication using SC modulation are illustrated in Table 1. Phase tracking for each communication method was based on the second-order PLL [35]. Adaptive equalizers used in the experiment were linear decision-direction equalizers and the sample interval was equal to the symbol interval T. Each information packet individually for two communication methods was flanked by a start linear frequency modulation (LFM) chirp marker used for packet synchronization. The LFM chirp was a 50 ms, 7.5–15.5 kHz chirp with a Hamming window. A guard time interval was set between the LFM chirp and the information packet to avoid interference, and the duration of which was 100 ms. Figure 7 illustrates the frequency spectrums of information packets respectively for two communication methods in the experiment.

#### 3.2.2. Experimental Results

**Figure 8:**
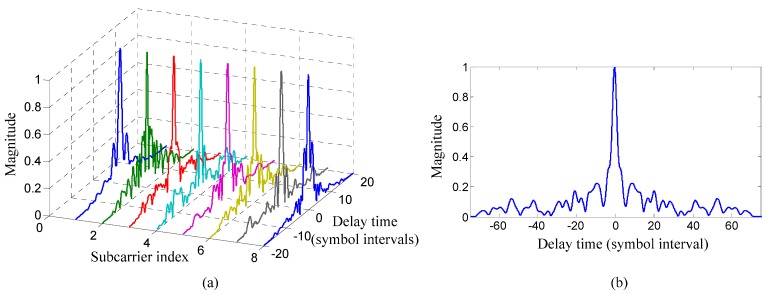
Comparison of each estimated composite channel from the transmit filter to the matched filter for the TR process between two communication methods in the experiment: (**a**) the proposed method; and (**b**) the TR acoustic communication using SC modulation.

Figure 8 shows the comparison of each estimated composite channel from the transmit filter to the matched filter for the TR process between the proposed method and the TR acoustic communication using SC modulation. The channel estimation was carried out based on the training symbols when the input signal-to-noise ratio (SNR) was 23 dB. The adaptive algorithm for channel estimation was RLS algorithm and the forgetting factor was 0.999. Figure 8 shows that the ISI span of the composite channel decreases from about 60 symbols to around eight symbols using the proposed method compared with the TR acoustic communication using SC modulation.

**Figure 9:**
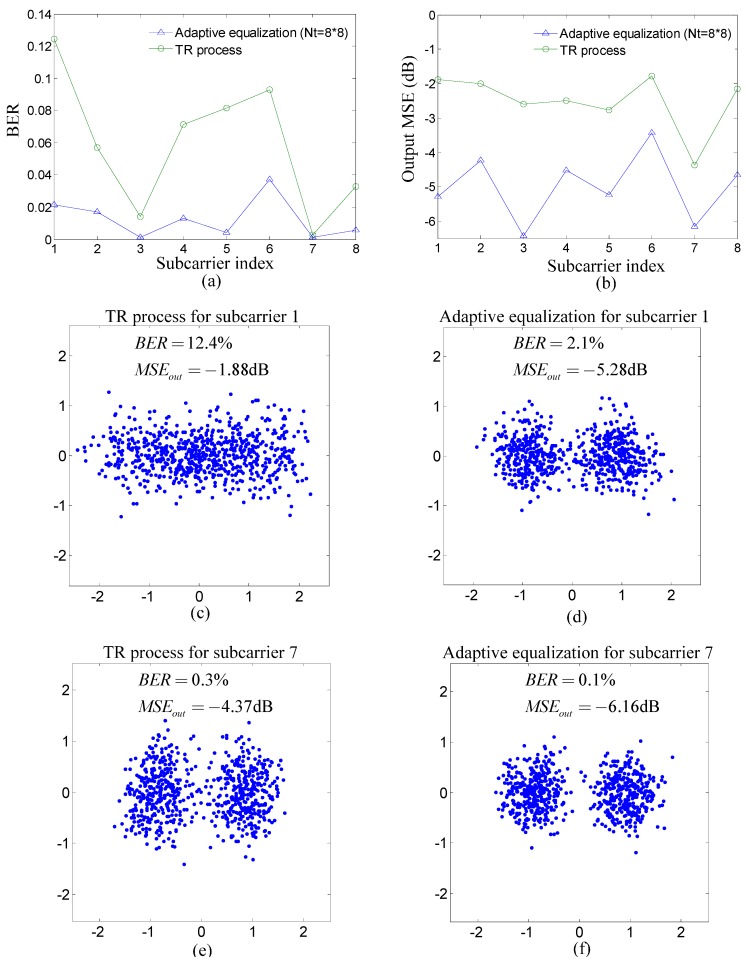
Experiment results for each subcarrier of the proposed method: (**a**) BER of the TR process and adaptive equalization; (**b**) output MSE of the TR process and adaptive equalization; (**c**,**d**) scatterplots of the TR process and adaptive equalization for subcarrier one; and (**e**,**f**) scatterplots of the TR process and adaptive equalization for subcarrier seven.

Figure 9 summarizes experiment results for each subcarrier of the proposed method. The first row of Figure 9 shows the performance of the TR process and adaptive equalization for each subcarrier where BER and the output MSE are used as performance indicators. It is noticed that the BER of the TR process for subcarrier one is the highest and that of subcarrier seven is the lowest. To demonstrate performance visually, the scatterplots for subcarriers one and seven are shown in the second row and the third row of Figure 9. The dots in each scatterplot denote the output symbols of the TR process or adaptive equalization, which should locate at −1 and 1 for BPSK without noise and ISI. Therefore, the scatterplots in Figure 9 further confirm that the performance of subcarrier seven is better than that of subcarrier one.

**Figure 10:**
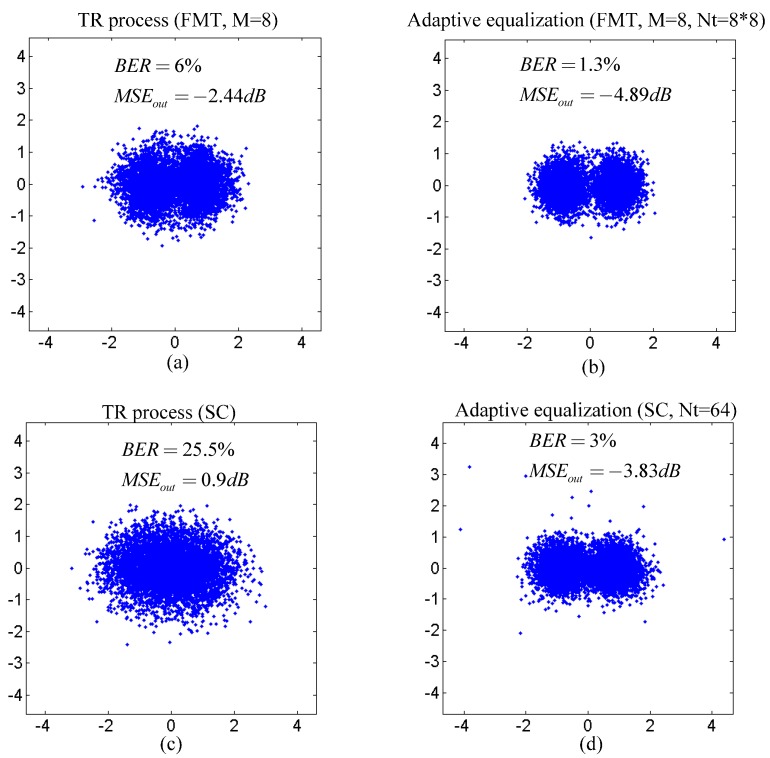
Performance comparison between two communication methods in the experiment: (**a**,**b**) performance of the TR process and adaptive equalization in the proposed method; and (**c**,**d**) performance of the TR process and adaptive equalization in the TR acoustic communication using SC modulation.

Figure 10 shows the overall performance of the proposed method and the TR acoustic communication using SC modulation. In Figure 10, the BER and output MSE of the proposed method are both the average over all subcarriers, and the dots in scatterplots for the proposed method correspond to the output symbols for all subcarriers. It is noticed that the performance of the TR process is improved using the proposed method compared with the TR acoustic communication using SC modulation. Moreover, when the total number of taps $N_{t}$ is fixed and the performance of adaptive equalization for post-process in two communication methods is relatively close, the computational complexity of adaptive equalization is cut by 7/8 using the proposed method compared with the TR acoustic communication using SC modulation. The experiment results are quite consistent with the simulation results.

## 4. Conclusions

When the symbol rate of the TR acoustic communication using SC is high, the large ISI span caused by multipath in underwater acoustic channels reduces the performance of the TR process and needs to be removed through long adaptive equalizers. To decrease the extent of ISI, a TR acoustic communication method using FMT modulation is proposed in the paper. The performance of the proposed method has been assessed through simulation and experiment, and compared with that of the TR acoustic communication using SC modulation. Results indicate that the ISI span of the proposed method is smaller than that of the TR acoustic communication using SC modulation, and therefore the proposed method can improve the performance of the TR process and reduce the computational complexity of adaptive equalization for post-process in contrast with the TR acoustic communication using SC modulation.
